# Atypical (Symplastic) Leiomyoma Arising in Pararectal Endometriosis Associated With Metastatic Ovarian Clear Cell Carcinoma: A Case Report

**DOI:** 10.1155/crip/7026230

**Published:** 2025-09-08

**Authors:** Nicholas Boivin, Ciaran Mannion

**Affiliations:** ^1^Hackensack Meridian School of Medicine, Nutley, New Jersey, USA; ^2^Department of Pathology, Hackensack Meridian University Hospital, Hackensack, New Jersey, USA

**Keywords:** atypical leiomyoma, bizarre leiomyoma, clear cell carcinoma, endometriosis, endomyometriosis, leiomyoma, leiomyoma with bizarre nuclei, ovarian fibroma, pleomorphic leiomyoma, symplastic leiomyoma

## Abstract

While very commonly encountered in the uterus, in particular in the wall of the uterine corpus, leiomyomas are far less frequently found in extragenital sites in females. Atypical leiomyomas (synonyms: pleomorphic leiomyomas, leiomyomas with bizarre nuclei, bizarre leiomyomas, or symplastic leiomyomas) are a rare subtype of leiomyomas, characterized by cells with bizarre, pleomorphic-appearing nuclei without (or with extremely low) associated mitotic activity. Despite the cytologic appearance, such tumors have a benign clinical course. Although there are cases of leiomyomas appearing outside the uterus in a background of endometriosis, cases specifically of atypical leiomyomas in this setting are exceptionally rare. We present a case of an atypical leiomyoma arising from pararectal endometriosis in a patient presenting with endometriosis-associated primary ovarian clear cell carcinoma, metastasis to the bladder peritoneum, and a peri-intestinal lymph node, in addition to a concurrent ovarian fibroma.

## 1. Introduction

Malignant transformation and smooth muscle metaplasia in the setting of endometriosis are both uncommon findings [1, 2]. Indeed, to the best of our knowledge, only a few cases of extrauterine atypical leiomyomas (synonyms: pleomorphic leiomyomas, leiomyomas with bizarre nuclei, bizarre leiomyomas, or symplastic leiomyomas) arising in a background of endometriosis have been reported [3]. Herein, we describe an interesting patient presentation with primary ovarian clear cell carcinoma, arising in a background of ovarian endometriosis, a concurrent ovarian fibroma, metastatic clear cell carcinoma to the bladder peritoneum and peri-intestinal lymph node, and an atypical leiomyoma arising in pararectal endometriosis.

## 2. Case Presentation

A 58-year-old female presented to the hospital with progressively worsening, nonradiating abdominal pain over the preceding 12 days. Her past medical history included uterine fibroids, ovarian cysts, and endometriosis. Initially, she only felt discomfort during urination; however, the pain became progressively more frequent, with no identifiable triggering or remitting factors. Prior to her presentation to the hospital, the patient had a very uncomfortable episode about 1 month earlier that had persisted for 8 days and for which her primary care doctor had prescribed baclofen (a muscle relaxant). Although she reported that the latter had alleviated some of her discomfort on that occasion, the baclofen failed to ameliorate the pain associated with the recurrent abdominal discomfort, resulting in her presentation to the hospital.

**Figure 1:**
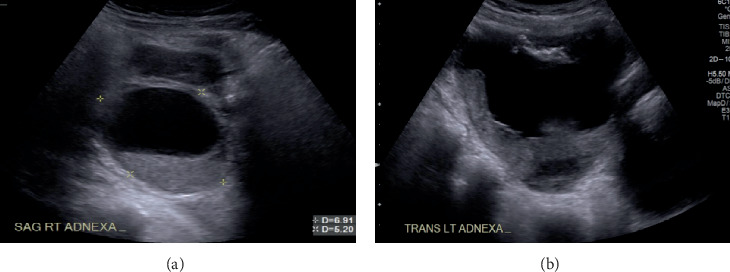
Pelvic ultrasound. (a) There is a mixed cystic and solid mass with irregular mural nodules and papillary projections measuring approximately 6.4 × 5.7 × 5.5 cm in the right adnexa. (b) There is another mixed cystic and solid mass with irregular mural nodules and papillary projections measuring approximately 8.8 × 11.4 × 8.5 cm in the left adnexa.

**Figure 2:**
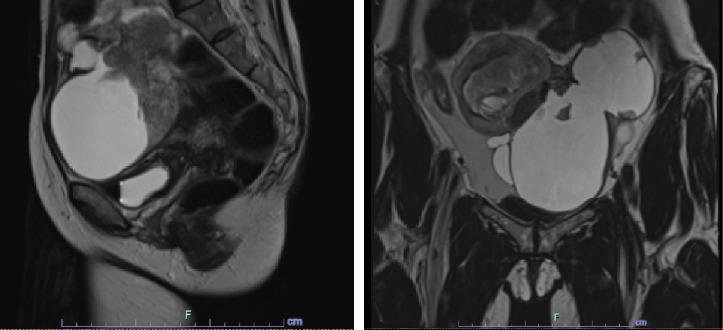
Magnetic resonance image showing an enlarged and leiomyomatous uterus measuring approximately 7.5 × 0.6 × 5.3 cm. There is also a large pelvic mass that is cystic and solid appearing measuring approximately 9.7 × 15.8 × 12.2 cm. The left and right ovaries cannot be distinguished on this exam.

A pelvic ultrasound revealed bilateral adnexal masses that were deemed suspicious for epithelial neoplasms, as well as multiple uterine fibroids (Figure 1). A subsequent abdominal MRI with and without contrast confirmed a large solid and cystic pelvic mass suspicious for ovarian carcinoma (Figure 2).

Surgical resection was performed. Inspection of the patient's pelvis revealed a large mass that was adherent to multiple loops of bowel and to the uterus. After separating the mass from the bowel, it was determined that the mass was a conglomeration of bilateral ovarian masses. A tumor deposit was found and removed along the left bladder pillar. Implants/deposits were also found and removed from the tissues adjacent to the sigmoid colon and rectum.

Gross examination revealed that both ovaries and fallopian tubes had been subsumed into a large cystic mass, measuring 15.5 × 8.0 × 4.0 cm and weighing 224 g. The outer surface was rough and irregular. Upon sectioning, the inner lining of the cystic cavity appeared tan in color and had a variably solid, cystic, and papillary architecture. Of note, however, a separate, smaller, morphologically distinct, well-circumscribed, tan, fibrous nodule, measuring 2.0 × 1.0 × 1.0 cm, was identified in the remaining ovarian tissue. The bladder implant appeared grossly as soft, tan, hemorrhagic tissue, measuring 3 × 2 × 1.5 cm. The sigmoid colon implant was yellow–gray and measured 1 × 0.8 × 0.6 cm. The pararectal implant was submitted as two fragments of tan-gray, rubbery soft tissue, measuring 0.7 and 1.2 cm in respective maximum dimensions.

**Figure 3:**
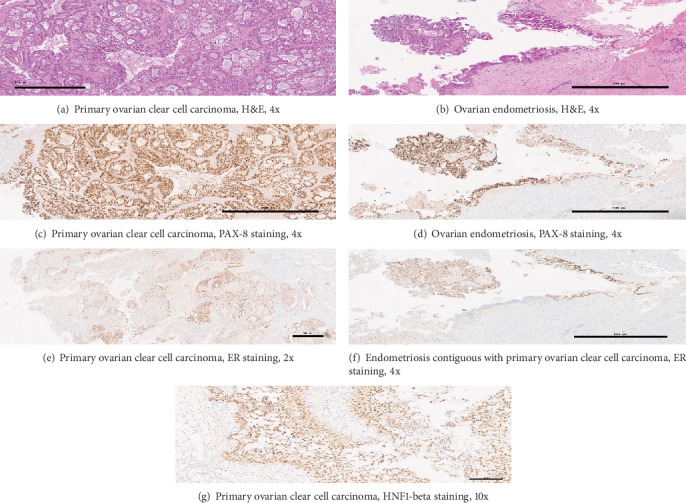
Histologic examination of the larger ovarian mass. (a) H&E at 4x power shows cells with clear cytoplasm, atypical hobnail nuclei, and a tubulocystic architecture, consistent with a clear cell carcinoma. (b) H&E from a different cystic area of the same mass at 4x power showing contiguous background endometriosis. (c) Immunohistochemical staining for PAX-8 at 4x power reveals strong nuclear staining in the area of clear cell carcinoma. (d) Similarly, PAX-8 staining at 4x power is more strongly expressed in the clear cell carcinoma, with discernible but weaker intensity nuclear staining in the contiguous background endometriosis. (e) Immunohistochemical staining for ER at 2x power with scattered nuclear staining in the area of clear cell carcinoma. (f) Stronger intensity ER staining at 4x power is evident in the area of endometriosis. (g) Strong, widespread nuclear staining for HNF1-beta in the area of clear cell carcinoma at 10x power.

**Figure 4:**
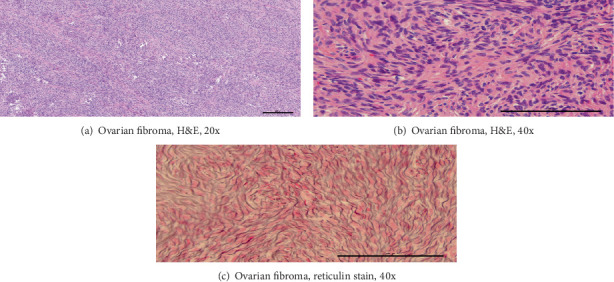
Histologic examination of the ovarian fibroma. (a) H&E at 20x power shows spindle cells without appreciable variation in nuclear size or cytologic atypia. (b) H&E at 40x power reaffirms the cytologically bland nature and relatively uniform appearance of the spindle cells, without discernible mitotic activity. (c) Reticulin stain at 40x power highlights investment around individual cells.

**Figure 5:**
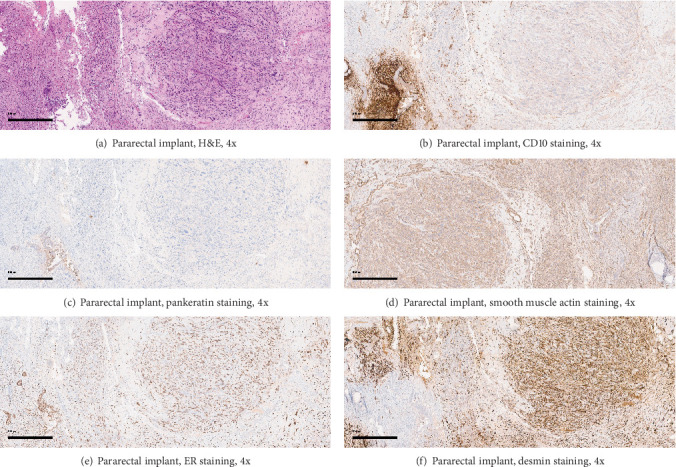
Microscopic images from the pararectal implant, all taken at 4x power. (a) H&E stain showed a nodular spindle cell lesion with variably sized nuclei centrally and to the right, with endometrial glands and stroma in the lower left-hand corner. (b) Immunohistochemical staining for CD10 shows strong stromal staining in the region with endometrial stroma. (c) Pankeratin staining is evident in the endometrial glands within the endometriosis, but not in the atypical spindle cell nodule. (d) Smooth muscle actin is positive in the spindle cells, but not in the endometrial glands. (e) Strong ER staining is apparent in both the endometriosis and in the atypical spindle cell nodule, (f) whereas desmin is strongly expressed in the spindle cells, but not in the endometrial glands or stroma of the endometriosis.

**Figure 6:**
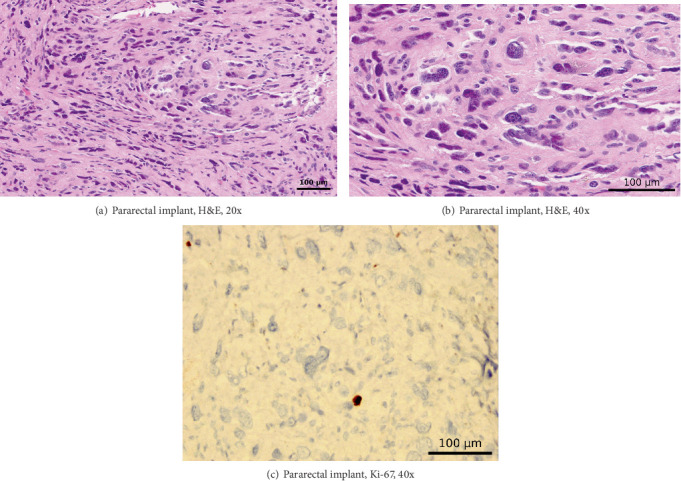
Microscopic images from the pararectal implant. The cells demonstrate marked nuclear cytologic atypia, but without accompanying mitotic figures. (a) H&E at 20x power. (b) H&E at 40x power. (c) Ki-67 staining at 40x power confirms the extremely low proliferation index in the cells with atypical, enlarged, and irregular bizarre nuclei.

Microscopic examination of the larger ovarian mass showed malignant cells with clear cytoplasm, atypical hobnail nuclei, and a tubulocystic architecture, reflective of a clear cell carcinoma (Figure 3a). In contrast, the second, smaller, circumscribed ovarian nodule had uniform spindled cells without necrosis, appreciable mitotic figures, or significant nuclear atypia (Figure 4a). Examination of the bladder implant and sigmoid implant revealed metastatic clear cell carcinoma to the bladder serosa and to a peri-intestinal lymph node. In contrast, the pararectal lesion consisted predominantly of a nodular arrangement of spindle cells with marked nuclear atypia but without readily discernible mitotic figures or lesional necrosis. Of note, the spindle cell nodule was not contiguous with the intestinal wall smooth muscle but rather was arising in association with benign endometrial glands and stroma, confirmatory of pararectal endometriosis (Figures 5a, 6a, and 6b).

Immunohistochemical (IHC) staining performed on the larger ovarian mass showed strong nuclear staining for HNF1-beta and PAX-8 (Figure 3c,g), variable intensity cytoplasmic staining with napsin A, and only scattered, weak-to-moderate intensity nuclear staining with estrogen receptor (ER) (Figure 3e), consistent with a clear cell carcinoma. In some sections, the tumor was contiguous with foci of benign endometriosis showing strong nuclear staining for PAX-8 and ER (Figures 3b, 3d, and 3f).

The second, smaller, circumscribed, fibrous, nodular, ovarian mass demonstrated strong nuclear staining for WT1 (Figure 4b), strong cytoplasmic staining with vimentin, variable staining with CD56, and no appreciable staining with HNF1-beta or napsin A. A reticulin stain showed investment of individual lesional cells. A Ki-67 stain confirmed a very low proliferation index. Collectively, these findings were confirmatory of an ovarian fibroma.

A panel of IHC stains on sections of the pararectal lesion revealed strong staining of the lesional atypical spindle cells for vimentin, smooth muscle actin, desmin, ER, CD10, and h-caldesmon (Figures 5b, 5d, 5e, and 5f). Pankeratin staining highlighted the adjacent endometriosis, without appreciable staining of the atypical spindle cell lesion (Figure 5c). No spindle cell staining for CD34 was observed and, in keeping with the H&E findings, the Ki-67 stain confirmed a very low proliferation index (Figure 6c). Collectively, the cytomorphologic features and IHC profile of the pararectal spindle cell lesion were consistent with a diagnosis of atypical leiomyoma (synonyms: pleomorphic leiomyoma, leiomyoma with bizarre nuclei, bizarre leiomyoma, or symplastic leiomyoma), arising in a setting of endometriosis.

## 3. Discussion

The biologic phenomenon of endometriosis is still incompletely understood. The two most widely held theories are that detached endometrial tissue from the uterine corpus is regurgitated through the fallopian tubes, spreading to other tissues (most often, intraabdominal) or implants in the cervix or elsewhere in the lower female genital tract, and/or that endometriosis may result from the transformation of a multipotential subepithelial stem cell population, in particular within, but not exclusive to, mullerian tissues [1, 4–7]. Although endometriosis is a common diagnosis, malignant transformations within foci of endometriosis are relatively infrequent, with an incidence rate of approximately 1% [1]. While the strongest malignant transformation association is with clear cell carcinoma, any malignancy encountered in the uterus may arise from endometriosis, including endometrioid carcinoma and serous carcinoma, as well as exceptionally rare cases of leiomyosarcoma arising in endometriosis [4, 8, 9]. The majority of these cases have been reported in the ovary [10].

It is possible for endometrial tissue to undergo smooth muscle metaplasia and result in the formation of a uterus-like mass [2]. This is referred to as endomyometriosis or extrauterine adenomyoma and can result in a setting of endometriosis or after uterine surgery. The frequency of smooth muscle metaplasia occurring in endometriosis is not well documented, but one study found that out of 265 patients with endometriosis, none of them had evidence of a leiomyoma arising from endometriosis [2]. Cases of endomyometriosis are sparsely reported, with a recent literature review identifying 85 cases [11]. While the large majority of endomyometriosis cases occur in the ovary, there have been case reports of uterus-like masses at other sites, including the small bowel mesentery, broad ligament, conus medullaris, liver, bowel wall, and sigmoid mesocolon [7, 11, 12]. Of note, endomyometriosis has also rarely been reported to occur in males in the paratestis, scrotum, and bladder serosa [5]. There have been nine reported cases of endomyometriosis with a concurrent malignancy [11]. Examples include a case of endomyometriosis with an associated clear cell carcinoma in the pelvic retroperitoneal area and a case of clear cell carcinoma occurring in the broad ligament that was reported to be arising from an adenomyoma in the setting of endometriosis [13, 14].

Uterine leiomyomas are common, with an incidence of almost 40% in women over age 50 years [15]. In addition to the more conventional, relatively uniform appearance of the vast majority of such leiomyomas, numerous other subtypes of leiomyomas, such as cellular, myxoid, leiomyomas with hydropic degeneration, epithelioid leiomyomas, and atypical leiomyomas (synonyms: pleomorphic, bizarre, or symplastic leiomyomas), are well documented and occasionally encountered, but these subtypes of leiomyomas have an estimated prevalence of just 0.23% [16]. Given their infrequency and variance in appearance from more conventional leiomyomas, such subtypes can, on occasion, pose a diagnostic challenge and are more apt to be mistaken for malignancies. This case presents an example of an atypical leiomyoma (synonyms: pleomorphic leiomyoma, leiomyoma with bizarre nuclei, bizarre leiomyoma, or symplastic leiomyoma), arising in a setting of pararectal endometriosis.

Despite the high frequency of leiomyomas and endometriosis in the general population, it is somewhat surprising that reported cases of leiomyomas, even of more conventional leiomyomas, arising in the setting of endometriosis are rare, let alone a case of an atypical leiomyoma. In a series of 54 ovarian smooth muscle tumors, largely retrieved from the consultation files of Dr. Robert Scully at Massachusetts General Hospital (MGH), only four cases of ovarian smooth muscle tumors associated with coexisting ovarian endometriosis were encountered [9]. Furthermore, in that entire collection, there was only one reported case of an atypical leiomyoma, and the latter neither occurred in a setting of endometriosis nor did that patient have a history of endometriosis [9, 17].

Cases of other leiomyoma subtypes and smooth muscle tumors of uncertain malignant potential occurring in a background of endometriosis appear to be equally rare [9, 18]. There have also been reports of nodular smooth muscle metaplasia occurring in peritoneal endometriotic foci and leiomyomatosis peritonealis disseminata coexisting with endometriosis within the same lesion [19, 20]. It should also be acknowledged that it can be challenging to track down all such cases reported due to the range of terminology used to describe coexistent benign endometrial glands and endometrial stroma with a concurrent extrauterine smooth muscle tumor (endomyometriosis vs. extrauterine mass vs. extrauterine adenomyoma vs. uterus-like mass).

In summary, to the best of our knowledge, the case presented herein is unique with respect to the concurrence of a pararectal atypical leiomyoma (synonyms: pleomorphic leiomyoma, leiomyoma with bizarre nuclei/bizarre leiomyoma, or symplastic leiomyoma) arising in a background of pararectal endometriosis, primary ovarian clear cell carcinoma arising from ovarian endometriosis with metastasis to bladder serosa and a peri-intestinal lymph node, and an ipsilateral ovarian fibroma.

## Data Availability

Data sharing is not applicable to this article as no datasets were generated or analyzed during the current study.
